# Supplementary material for: Chromothripsis during telomere crisis is independent of NHEJ, and consistent with a replicative origin
Source: Genome Res. 2019 May;29(5):737–49. doi: 10.1101/gr.240705.118 (PMC6499312; doi:10.1101/gr.240705.118)
Supplement: Supplemental Material [file supp_gr.240705.118_Supplemental_file_1.zip › contigs/annotated_contigs/DB77/contig.2.DB77_length_706_mean_cov_11.5694050992.docx]

**DB77_length_706_mean_cov_11.5694050992**

```
TCCCAACAATGAACTCGAGGAATTTTAGAAACCCAGTAGAATACTCTAACATGAGAATTTGTATTATTTAAATCTTGAATACCAGAACC
 >chr4:33661306-33661595 - E=4e-159
AAAGATTAGAGGAGTTCTGTTTGTTTGTTTTTGTATTTTGAGACAGAATTTCACTCTTGTTACCCAGCCTACAGGGCAATGGCGTTATG

TTGACTCACTGCAACCTCCGCCTCCCAGGTTCAAGTGATTCTCCTGCCTCAGCCTCCTGAGTAGCTGGGATTACAGGTGACCACCAGCA

CACCCTGCATATATATATAT|AT|GTACTTAGTGTCCTAGATTAAGCATTAATTTTTCTACTGAATCACAGGTATAAGTCCTGAGCTAC
                     >chr4:33641430-33641849 + E=2e-240
TACTAGAGAAAGACAGTTTTTAGCAAAACAAACAAACAAACAAACAAAAAAAACAGGGAGCATAGCTTTGCTCCAACACTCTAAGAATC

TGGACTATGATTTTCCCAGTAGATTTTTTGAGAGACTCCATATAGTCTTCTTATCTGCTGTAGATTCATCATCAAATACCATTGTGTCC

ACTAATCGTAAGGACAGAACATCTGGACCTGCTCGAGGCATTCCTTCCTCATCCTTATTTAATGTTGGAGACCACATCGATTAATAGGT

AGCTGATGAGAATCCCATCCATATGCCAAATTTGTTACATCCTAAATTCAAAGATTCCCCTCAATTCTCATTATGTGCAGTGGAT
```
